# Comparing the Functional Outcomes of Hyaluronic Acid Injection and Dry Needling in Lateral Epicondylitis: A Retrospective Study

**DOI:** 10.7759/cureus.84732

**Published:** 2025-05-24

**Authors:** Richik Sarkar, Gils Thampi, Nagakumar JS

**Affiliations:** 1 Orthopaedics, Sri Devaraj Urs Academy of Higher Education and Research, Kolar, IND

**Keywords:** dry needling, elbow tendinopathy, functional outcomes, hyaluronic acid, lateral epicondylitis, pain management

## Abstract

Background

Lateral epicondylitis (LE), commonly referred to as tennis elbow, is a widespread condition characterized by elbow pain and functional impairment. Conservative treatment options include physiotherapy, pharmacotherapy, and interventional procedures such as dry needling (DN) and hyaluronic acid (HA) injections. HA, an emerging treatment, has shown anti-inflammatory and lubricating properties, which may enhance tendon healing. However, direct comparative evidence between HA and DN injections for the management of LE remains limited. The main objective of this research was to compare the functional outcomes of HA injections versus DN in patients with LE, specifically focusing on pain relief, and overall functional improvement over a six-month period.

Materials and methods

A retrospective comparative study was conducted at a tertiary care institution between June and November 2024, involving 64 patients diagnosed with LE. The patients were divided into two equal groups: Group A (hyaluronic acid injection, n=32) received a single-dose hyaluronic acid injection under ultrasound guidance, followed by rehabilitation, while Group B (dry needling, n=32) underwent peritendinous dry needling using a 25G needle. Pain reduction was assessed using the Visual Analog Scale (VAS) and functional improvement was evaluated using the Quick Disabilities of the Arm, Shoulder, and Hand (QuickDASH), Oxford Elbow Score, Mayo Elbow Score, and Patient-Rated Tennis Elbow Evaluation (PRTEE) at baseline, post-procedure, first month, third months and sixth months. Statistical analyses were performed using SPSS version 22 (IBM Corp, Armonk, NY).

Results

At six months, HA injections demonstrated a significantly greater reduction in pain, as measured by the VAS (2.41±0.50 vs. 3.44±0.56, p < 0.001), and superior functional outcomes in the QuickDASH (19.03±2.93 vs. 23.44±3.64, p<0.001), the Oxford Elbow Score (91.87±5.89 vs. 82.13±1.52, p<0.001), and the Mayo Elbow Score (91.75±6.34 vs. 82.41±2.00, p <0.001). The complication rates were lower in the HA group, with significantly less local tenderness (3.1% vs. 25.0%).

Conclusion

Both HA injection and DN effectively improved pain and function in LE patients, but HA demonstrated superior long-term efficacy and safety. Future studies with larger sample sizes and extended follow-up periods are recommended.

## Introduction

Lateral elbow tendinopathy, also known as lateral epicondylalgia, lateral epicondylitis, or tennis elbow, is a common source of pain and disability in the elbow [1,2]. It occurs in approximately 1%-3% of the general population and 7% of manual laborers, typically impacting individuals aged 42-54 years, with no significant sex predilection [3]. Currently, various conservative treatment options are available for lateral epicondylitis, including physiotherapy, pharmacotherapy, and interventional physiatry [4]. Among the interventional procedures, periarticular hyaluronic acid (HA) injection is an emerging treatment option, although it lacks robust evidence to support its widespread use. The choice of treatment largely depends on the patient’s clinical needs and preferences, the clinician’s experience, and current evidence [4]. HA injection for lateral epicondylitis shows promising results due to its anti-inflammatory and lubricating properties [5-7]. HA is the primary component of synovial fluid, providing lubrication and shock absorption for arthritic joints. It has the potential to: (a) reduce surface friction between tendons; (b) prevent the influx of inflammatory mediators; (c) promote analgesia by desensitizing nociceptors; and (d) upregulate vascular endothelial growth factor and type IV collagen, thereby accelerating tendon healing, among other benefits [5,7]. Other conservative treatments for lateral elbow tendinopathy, such as needling therapies (dry needling and acupuncture), have recently gained popularity in clinical settings.

Dry needling (DN), another minimally invasive intervention, involves the insertion of thin needles into soft tissues at specific points. In the context of lateral epicondylitis, DN targets myofascial trigger points and peri-tendinous tissue by provoking a localized healing response through microtrauma. This technique is believed to disrupt dysfunctional muscular activity, promote blood flow, and facilitate endogenous repair mechanisms, and it has grown in clinical popularity as an option for managing chronic tendinopathies, including tennis elbow.

Despite the increasing use of both HA injections and DN, direct comparative studies evaluating their efficacy and safety for managing lateral epicondylitis remain limited. While each method shows promise for pain reduction and functional improvement, head-to-head investigations are scarce and leave unanswered questions regarding optimal long-term management strategies for this common condition.

The present study addresses this gap by retrospectively comparing the functional outcomes and complication rates of HA injection and DN in patients with lateral epicondylitis. The primary aim is to evaluate and contrast pain relief and overall functional improvement at multiple time points over a six-month follow-up period, thereby helping to inform evidence-based treatment choices for clinicians and patients alike.

## Materials and methods

A hospital-based retrospective study was conducted involving 64 patients treated with either HA injection or DN for lateral epicondylitis at R.L. Jalappa Hospital in Tamaka, Kolar, Karnataka, India, from June 2024 to November 2024. Ethical clearance was obtained from the Institutional Ethical Committee of Sri Devaraj Urs Medical College (approval number SDUAHER/R&D/CEC/SDUMC-PG/05/NF/-2025-2026) to conduct this research. All participants provided written informed consent.

In this research, patients aged 25-60 years with a clinical diagnosis of lateral epicondylitis persisting for more than six weeks and positive Cozen’s and Mills’ tests were included. Patients with a history of elbow surgery, rheumatoid arthritis, systemic inflammatory conditions, or corticosteroid injections within the past three months were excluded. Data retrieved from the patients’ medical records were thoroughly analyzed, with a minimum follow-up period of six months. The data were retrospectively evaluated to assess clinical and functional outcomes, including pain reduction measured using the Visual Analog Scale (VAS) at baseline, post-procedure, first month, third month and sixth month; functional outcomes recorded through the Quick Disabilities of the Arm, Shoulder, and Hand (QuickDASH) [7], Oxford Elbow Score [8], Mayo Elbow Score [9], and Patient-Rated Tennis Elbow Evaluation (PRTEE) [10] at baseline, post-procedure, first month, third month and sixth month; and complications such as local tenderness, stiffness, infection, or worsening symptoms.

The total sample size was 64, divided equally into two groups: Group A (HA injection, n=32) received a single-dose HA injection (1 mL, 15mg/mL concentration) directly at the lateral epicondyle. The injection was performed under ultrasound guidance to ensure accurate localization. Following the injection, patients were advised to rest the affected arm for two to three days before beginning a post-injection rehabilitation protocol that included progressive strengthening exercises. Group B (dry needling, n=32) underwent a single session of peritendinous dry needling with a 25G needle. The procedure aimed to induce localized bleeding and microtrauma, thereby stimulating natural healing responses. After the needling, patients underwent supervised stretching exercises and were instructed on a gradual return to activity. All injection and needling procedures were done by qualified musculoskeletal specialists. The sample size was calculated based on the difference in mean VAS scores between the HA and DN groups from a study by Saornil JV et al., which reported scores of 6.85±1.20 and 6.00±1.10, respectively [11]. Using a 95% confidence limit and 80% power, a sample size of 29 per group was estimated using MedCalc software and the formula: \( N = \frac{2SD^2 \left( Z_{\alpha/2} + Z_{\beta} \right)^2}{d^2} \), where SD was 1.15, Zα/2 was 1.96, Zβ was 0.842, and d was 0.85 [12]. Accounting for a 10% nonresponse rate, the final sample size was rounded to 32 cases per group.

Data were entered into a Microsoft Excel (Microsoft, Redmond, WA, USA) spreadsheet and analyzed using SPSS version 22 (IBM Corp, Armonk, NY, USA) and Epi Info version 7.2.1 (CDC Atlanta, Atlanta, GA, USA). Categorical data were represented as frequencies and proportions. The Chi-square test was employed as a significant test for qualitative data. Continuous data were expressed as means and standard deviations. The normality of the continuous data was assessed using the Kolmogorov-Smirnov test and the Shapiro-Wilk test. An independent t-test was utilized to determine the mean difference between two quantitative variables, while the Mann-Whitney U test was applied for non-parametric data between two groups. A p-value of <0.05 was considered statistically significant, assuming that all necessary conditions for statistical tests were met. The statistical software used for data analysis included Microsoft Excel and SPSS version 22 [13-15].

## Results

In the present study, the majority of subjects in the HA group were aged between 41 and 50 years (40.6%), while the majority in the DN group also fell within the same age range (34.4%). The mean age in the HA group was 40.13±10.127 years, compared to 41.91±11.869 years in the DN group. There was no significant difference in age distribution between the two groups (p=0.521). Regarding sex distribution, 75.0% of subjects in the HA group were male, compared to 62.5% in the DN group, with no significant difference observed (p=0.281). The distribution of side involvement (left or right) and diagnosis (L-LE or R-LE) also showed no significant differences (p=0.316 for both). Comorbidities such as diabetes mellitus (DM), hypertension (HTN), and the combination of DM/HTN were similarly distributed, with no significant differences noted (p=0.860) (Table 1).

**Table 1:** Profile of subject’s comparison between two groups (n = 64)

Characteristics		Group				P value
		Hyaluronic Acid (n=32)		Dry Needling (n=32)		
		Count	%	Count	%	
Age	<30 years	7	21.9%	9	28.1%	0.588
	31 to 40 years	8	25.0%	5	15.6%	
	41 to 50 years	13	40.6%	11	34.4%	
	51 to 60 years	4	12.5%	7	21.9%	
	Mean ± SD	40.13 ± 10.127		41.91 ± 11.869		0.521
Sex	Female	8	25.0%	12	37.5%	0.281
	Male	24	75.0%	20	62.5%	
Diagnosis	Left LE	17	53.1%	13	40.6%	0.316
	Right LE	15	46.9%	19	59.4%	
Comorbidities	DM	4	12.5%	6	18.8%	0.860
	DM/HTN	3	9.4%	3	9.4%	
	HTN	2	6.2%	1	3.1%	
	No	23	71.9%	22	68.8%	

The mean pre-treatment VAS score was 7.90±0.37 in the HA group and 7.86±0.36 in the DN group, showing no significant difference between the groups (p=0.645). Immediately post-procedure, the scores were 7.78±0.38 for the HA group and 7.80±0.37 for the DN group (p=0.824), remaining statistically similar. At one month, the scores decreased to 6.00±0.76 in the HA group and 5.59±0.61 in the DN group (p=0.016). At three months, the scores were 4.38±0.75 and 4.47±0.62, respectively (p=0.668), indicating no significant difference. At six months, the HA group exhibited a significantly lower VAS score of 2.41±0.50 compared to 3.44±0.56 in the DN group (p<0.001), suggesting better long-term pain reduction (Table 2).

**Table 2:** Visual Analog Scale (VAS) comparison between two groups (n=64)

VAS Score	Group						P value
	Hyaluronic Acid (n=32)			Dry Needling (n=32)			
	Mean	SD	Median	Mean	SD	Median	
Pre-procedure	7.90	0.37	8	7.86	0.36	8	0.645
Post-procedure	7.78	0.38	8	7.80	0.37	8	0.824
1st Month	6.00	0.76	6	5.59	0.61	6	0.016
3rd Month	4.38	0.75	4	4.47	0.62	4	0.668
6th Month	2.41	0.50	2	3.44	0.56	3	<0.001

The mean pre-treatment QuickDASH score was 75.00±5.30 in the HA group and 75.56±5.28 in the DN group, with no statistically significant difference between the groups (p=0.670). Immediately post-procedure, the scores were 73.10±4.05 for the HA group and 73.44±3.98 for the DN group (p=0.772), remaining comparable. At one month, the scores were 61.03±3.78 and 62.53±6.48, respectively (p=0.201), with no significant difference. By three months, the scores were 47.50±4.09 for the HA group and 47.53±6.35 for the DN group (p=0.635). However, at six months, the HA group demonstrated significantly greater improvement, with a score of 19.03±2.93 compared to 23.44±3.64 in the DN group (p<0.001) (Table 3).

**Table 3:** QuickDASH score comparison between two groups (n=64)

QuickDASH Score	Group						P value
	Hyaluronic Acid (n=32)			Dry Needling (n=32)			
	Mean	SD	Median	Mean	SD	Median	
Pre-procedure	75.00	5.30	75	75.56	5.28	76	0.670
Post-procedure	73.10	4.05	73	73.44	3.98	73	0.772
1st Month	61.03	3.78	61	62.53	6.48	65	0.201
3rd Month	47.50	4.09	50	47.53	6.35	45	0.635
6th Month	19.03	2.93	17	23.44	3.64	22	<0.001

The mean pre-treatment Oxford Elbow Score was 61.50±2.45 in the HA group and 61.97±2.43 in the DN group, with no statistically significant difference between the groups (p=0.422). Immediately post-procedure, the scores were 65.30±4.80 for the HA group and 65.00±1.80 for the DN group (p=0.740), remaining comparable. At one month, the scores were 70.88±6.25 for the HA group and 72.75±2.00 for the DN group (p=0.058), indicating no significant difference. Similarly, at three months, the scores were 79.72±5.30 for the HA group and 78.44±1.48 for the DN group (p=0.100). However, at six months, the HA group demonstrated significantly greater improvement, achieving a score of 91.87±5.89 compared to 82.13±1.52 in the DN group (p<0.001) (Table 4).

**Table 4:** Oxford Elbow score comparison between two groups (n=64)

Oxford Elbow Score	Group						P value
	Hyaluronic Acid (n=32)			Dry Needling (n=32)			
	Mean	SD	Median	Mean	SD	Median	
Pre-procedure	61.50	2.45	61	61.97	2.43	62	0.422
Post-procedure	65.30	4.80	65	65.00	1.80	65	0.740
1st Month	70.88	6.25	70	72.75	2.00	73	0.058
3rd Month	79.72	5.30	79	78.44	1.48	78	0.100
6th Month	91.87	5.89	93	82.13	1.52	82	<0.001

The pre-treatment Mayo Elbow scores were comparable between the two groups, with values of 62.19±3.58 for one group and 63.31±1.23 for the other (p=0.793). At one month, the scores were 72.06±3.56 and 73.09±1.35, respectively (p=0.438). However, at three months, the HA group demonstrated a significantly higher score of 79.13 ± 4.84 compared to 76.84 ± 1.94 in the DN group (p<0.001). At six months, the HA group exhibited a notable improvement, achieving a score of 91.75 ± 6.34, while the DN group scored 82.41±2.00 (p<0.001) (Table 5).

**Table 5:** Mayo Elbow Score comparison between two groups (n=64)

Mayo Elbow Score	Group						P value
	Hyaluronic Acid (n=32)			Dry Needling (n=32)			
	Mean	SD	Median	Mean	SD	Median	
Pre-procedure	62.19	3.58	65	63.31	1.23	64	0.793
Post-procedure	62.19	3.58	65	63.66	1.93	64	0.989
1st Month	72.06	3.56	70	73.09	1.35	73	0.438
3rd Month	79.13	4.84	80	76.84	1.94	78	<0.001
6th Month	91.75	6.34	95	82.41	2.00	83	<0.001

The mean pre-treatment Patient-Rated Tennis Elbow Score was 75.10±5.20 in the HA group and 75.66±5.18 in the DN group, with no statistically significant difference between the groups (p=0.670). Immediately post-procedure, the scores were 73.30±4.05 for the HA group and 73.44±3.98 for the DN group (p=0.890), remaining similar. At one month, the scores were 61.56±3.60 and 62.78±6.83, respectively (p=0.315), again showing no significant difference. At three months, the scores were 48.03±4.08 for the HA group and 47.72±7.54 for the DN group (p=0.265). At six months, the scores were 20.34±3.96 and 22.56±2.63, respectively, also with no significant difference (p=0.195) (Table 6).

**Table 6:** Patient-Rated Tennis Elbow Score comparison between two groups (n=64)

Patient Rated Tennis Elbow Score	Group						P value
	Hyaluronic Acid (n=32)			Dry Needling (n=32)			
	Mean	SD	Median	Mean	SD	Median	
Pre-procedure	75.10	5.20	75	75.66	5.18	76	0.670
Post-procedure	73.30	4.05	73	73.44	3.98	73	0.890
1st Month	61.56	3.60	61	62.78	6.83	65	0.315
3rd Month	48.03	4.08	50	47.72	7.54	45	0.265
6th Month	20.34	3.96	20	22.56	2.63	22	0.195

The majority of participants in the HA group (84.4%) experienced no complications, compared to 62.5% in the DN group (p=0.09). The incidence of infection was identical in both groups (6.2%). Local tenderness was significantly more prevalent in the DN group (25.0%) compared to the HA group (3.1%). The incidence of stiffness was the same in both groups (6.2%) (Table 7).

**Table 7:** Complications comparison between two groups (n=64)

Complications	Group				P value
	Hyaluronic Acid (n=32)		Dry Needling (n=32)		
	Count	%	Count	%	
Nil	27	84.4%	20	62.5%	0.09
Infection	2	6.2%	2	6.2%	
Local Tenderness	1	3.1%	8	25.0%	
Stiffness	2	6.2%	2	6.2%	

## Discussion

The present study demonstrated that both HA and DN were effective in reducing pain and improving function in patients with lateral epicondylitis. However, HA exhibited superior long-term efficacy, as evidenced by significantly lower VAS and enhanced functional outcomes at the six-month follow-up. These findings are consistent with previous literature evaluating HA injections for chronic tendinopathies. Lynen [4] reported that HA injections significantly reduced pain and improved function in patients with tendinopathies, which aligns with our findings at the six-month mark. Similarly, Tosun et al. [5] found that HA was more effective than corticosteroids in treating lateral epicondylitis, further supporting its efficacy for sustained pain relief.

The mechanism underlying the long-term benefits of HA is likely attributed to its viscoelastic properties, which enhance lubrication, reduce inflammation, and promote tendon healing. This aligns with the findings of Flores et al. [6], who demonstrated the efficacy of peritendinous HA in alleviating pain associated with supraspinatus tendinopathy. In contrast, DN provides short-term pain relief primarily by disrupting myofascial trigger points and facilitating local healing through microtrauma [2]. Furthermore, research conducted by Saornil et al. [11] supports the notion that while DN is effective for pain relief, HA injections yield superior outcomes in cases of patellar osteoarthritis.

Previous research by Zinger et al. found that HA injections resulted in significant reductions in pain and improved function in patients with lateral epicondylitis, supporting our findings that HA provided better long-term pain relief [16]. Similarly, a study by Petrella et al. [17] demonstrated that HA enhanced tendon healing due to its anti-inflammatory and lubricating properties. These mechanisms may account for the superior VAS and functional scores observed at six months in our study.

In contrast, DN has been extensively studied for tendinopathies and musculoskeletal pain conditions. A meta-analysis by Yan et al. [18] concluded that DN effectively reduces pain and improves function in LE, particularly in the short term. However, conflicting results have been reported, as demonstrated by a study conducted by Uygur et al. [19], which found that DN provided only temporary relief compared to corticosteroid injections. Our findings align with this observation, as DN demonstrated rapid pain relief but was less effective than HA in achieving long-term outcomes.

Variability in results among studies may be attributed to differences in study design, patient demographics, injection techniques, and follow-up duration. The precise mechanism of HA in tendon healing remains under investigation; however, its role in promoting extracellular matrix synthesis and reducing inflammation likely contributes to sustained improvement. Additionally, the complications observed in our study are consistent with previous literature. HA demonstrated fewer adverse effects, while DN was associated with localized tenderness, as reported by Mishra et al. [20].

Despite significant improvements in VAS, QuickDASH, Oxford Elbow Score, and Mayo Elbow Score at six months, the Patient-Rated Tennis Elbow Score did not exhibit significant differences. This suggests that patients' perceptions of functional recovery may vary. These findings are consistent with those of Di Filippo et al. [1], who highlighted inconsistencies in functional assessments across LE studies. Luk et al. [3] proposed that chronic tendinopathies may require multimodal treatment, which could explain why the PRTEE did not demonstrate statistical significance in our study.

Complication rates were lower in the HA group, with significantly less local tenderness compared to the DN group, reinforcing the safety profile of HA. These findings align with previous reports indicating that HA is well tolerated with minimal adverse effects [4]. However, the variability in treatment responses across studies may arise from differences in injection techniques, patient demographics, and follow-up durations.

Overall, our results support the use of HA as an effective long-term treatment for lateral epicondylitis, demonstrating superior pain relief and functional improvements compared to DN. Future studies with larger sample sizes and extended follow-up periods are necessary to further validate these findings and optimize treatment protocols.

Limitations

This study has several limitations. Its retrospective design and small sample size may introduce selection bias and limit the statistical power of the findings. As a single-center study lacking randomization or blinding, the results may not be widely generalizable and are susceptible to observer bias. The six-month follow-up period does not capture long-term outcomes, and variations in patient adherence to rehabilitation protocols could influence the results. Another key limitation of this study is that information on patients’ use of analgesic or anti-inflammatory medications was not recorded, potentially confounding the effects of the interventions on pain and functional outcomes. Furthermore, reliance on subjective outcome measures and the exclusion of certain patient groups further restrict the applicability of the conclusions. Larger, prospective, multicenter studies with extended follow-up are necessary to validate these findings.

## Conclusions

In this retrospective comparison of hyaluronic acid (HA) injections versus dry needling (DN) for the management of lateral epicondylitis, both treatments effectively reduced pain and improved function. However, HA demonstrated superior long-term efficacy at six months, with significantly better VAS pain scores (2.41±0.50 vs. 3.44±0.56, p<0.001), functional outcomes including QuickDASH (19.03±2.93 vs. 23.44±3.64, p<0.001), Oxford Elbow Score (91.87±5.89 vs. 82.13±1.52, p<0.001), and Mayo Elbow Score (91.75±6.34 vs. 82.41±2.00, p<0.001). The HA group also experienced fewer complications, particularly local tenderness (3.1% vs. 25.0%). These findings suggest that the superior efficacy of HA is likely attributable to its viscoelastic, anti-inflammatory, and tissue-healing properties, making it a preferable option for the long-term management of lateral epicondylitis.
